# Quantitative assessment of choriocapillaris flow deficits in diabetic retinopathy: A swept-source optical coherence tomography angiography study

**DOI:** 10.1371/journal.pone.0243830

**Published:** 2020-12-11

**Authors:** Yining Dai, Hao Zhou, Qinqin Zhang, Zhongdi Chu, Lisa C. Olmos de Koo, Jennifer R. Chao, Kasra A. Rezaei, Steven S. Saraf, Ruikang K. Wang

**Affiliations:** 1 Department of Bioengineering, University of Washington, Seattle, Washington, United States of America; 2 Department of Ophthalmology, University of Washington, Seattle, Washington, United States of America; National Yang-Ming University Hospital, TAIWAN

## Abstract

**Purpose:**

To quantitatively assess choriocapillaris (CC) flow deficits in eyes with diabetic retinopathy (DR) using swept-source optical coherence tomography angiography (SS-OCTA).

**Methods:**

Diabetic subjects with different stages of DR and age-matched healthy subjects were recruited and imaged with SS-OCTA. The *en face* CC blood flow images were generated using previously published and validated algorithms. The percentage of CC flow deficits (FD%) and the mean CC flow deficit size were calculated in a 5-mm-diameter circle centered on the fovea from the 6×6-mm scans.

**Results:**

Forty-five diabetic subjects and 27 control subjects were included in the study. The CC FD% in diabetic eyes was on average 1.4-fold greater than in control eyes (12.34±4.14% vs 8.82±2.61%, *P* < 0.001). The mean CC FD size in diabetic eyes was on average 1.4-fold larger than in control eyes (2151.3± 650.8μm^2^ vs 1574.4±255.0 μm^2^, *P* < 0.001). No significant difference in CC FD% or mean CC FD size was observed between eyes with nonproliferative DR and eyes with proliferative DR (*P* = 1.000 and *P* = 1.000, respectively).

**Conclusions:**

CC perfusion in DR can be objectively and quantitatively assessed with FD% and FD size. In the macular region, both CC FD% and CC FD size are increased in eyes with DR. SS-OCTA provides new insights for the investigations of CC perfusion status in diabetes in vivo.

## Introduction

Diabetes mellitus (DM) is a disease affecting nearly all blood vessel types and sizes [1]. One of the most common microvascular complications of DM is diabetic retinopathy (DR), which is a leading cause of acquired vision loss globally [2]. Whereas retinal vasculature provides oxygen and nutrition to the inner retina, 90% of the blood from the ophthalmic artery enters into the choroidal vasculature to supply the outer retina [3]. However, the inherent difficulty in viewing the choroid in vivo impedes both fundamental research investigation and clinical evaluation of the pathophysiological alterations in diabetic choroids.

The advent of indocyanine green angiography (ICGA) allows for visualization of the choroid. Although fluorescein angiography (FA) is extensively used for the evaluation of DR, abnormalities on ICGA representing choroidal neovascularization, vascular leakage, choroidal aneurysms, and ischemia have been reported in imaging studies of the choroid in DM [4]. However, unlike the large and intermediate-sized choroidal vessels seen on ICGA, the choriocapillaris (CC) appears as a diffuse indistinct haze due to insufficient resolution [5]. The lack of an imaging modality capable of visualizing and quantifying CC perfusion in vivo has hindered our understanding of the role of CC in diabetic eye disease.

With the development of swept-source optical coherence tomography (SS-OCT) angiography (SS-OCTA), new perspectives and opportunities for visualizing and quantifying the CC have begun to evolve. With commercially available SS-OCTA systems, CC flow deficits (FDs) (or flow voids) have been examined and quantified in normal eyes [6, 7] and those affected by several chorioretinal diseases [8–10]. The FDs in CC indicate areas where there is a lack of CC blood flow or blood flow below the detectable threshold of the OCTA systems [11]. Using a commercially available SS-OCTA, we have observed a significant increase in CC FDs in diabetic eyes without DR [12]. A recent study employing SS-OCTA has further confirmed the increased CC FDs in both DM Type 1 and DM Type 2 even in the absence of clinical signs of DR [13]. Based on the potential involvement of the CC in diabetic microangiopathy and its important role in supplying the outer retina especially the macula, we aimed to explore the macular CC perfusion in diabetic eyes with DR using SS-OCTA.

## Methods

### Study population

We performed a prospective, cross-sectional study that adhered to the tenets of the Declaration of Helsinki and in accordance with the Health Insurance Portability and Accountability Act. The protocol was approved by the Institutional Review Board (IRB) of the University of Washington. Signed IRB-approved consent was obtained from each subject before SS-OCTA imaging. All participants underwent a comprehensive ophthalmic examination including slit-lamp examination and dilated funduscopy. Each subject was required to have a normal anterior segment examination with sufficiently clear media to allow imaging. Diabetic subjects were required to have a best-corrected visual acuity of 20/100 or better. Axial length measurements were performed using a noncontact biometry instrument (IOLMaster^®^ 500; Carl Zeiss Meditec Inc., Jena, Germany). The severity of DR was graded based on the International Clinical Diabetic Retinopathy and Diabetic Macular Edema Severity scale [14]. Eyes with other ocular pathology (including other chorioretinal pathology, glaucoma, uveitis, an axial length > 25.2 mm or < 23.2 mm) were excluded from the study. Other exclusion criteria included recent clinic-based procedure (laser capsulotomy, laser photocoagulation, intravitreal or sub-Tenon’s injections) in 90 days prior to enrollment; major intraocular surgery (including vitrectomy, cataract extraction, and scleral buckle) within the prior 12 months; and systemic diseases that might affect ocular blood flow, such as uncontrolled hypertension, systemic lupus erythematosus, anemia, leukemia, and known cardiovascular disease. We also excluded eyes in which the maximum retinal thickness exceeded 500 μm on macular SS-OCT scans since edematous tissue decreased the extent of light source penetration [15], which may result in large areas of shadowing on the *en face* CC images. Eyes with intraretinal or preretinal lesions (e.g. hemorrhages or hard exudates) with a total area larger than 4 mm^2^ (about 20% of the 5-mm-diameter circular area) on the *en face* CC image were also excluded since large areas of shadowing induced by these lesions would artificially reduce the global background signal of the *en face* CC image. The right eye was selected as the study eye for each subject unless inclusion criteria were not met.

### OCTA image acquisition

Each subject was scanned using a SS-OCTA instrument with 100 kHz A-line rate at 1060 nm (PLEX^®^ Elite 9000; Carl Zeiss Meditec Inc., Dublin, CA, USA). A 6×6-mm scan in the central macula was performed, consisting of 500 horizontal A-lines at 500 vertical locations with 2 repeated scans in each fixed location. The complex optical microangiography (OMAG) algorithm was used to obtain OCTA images [16]. Scans with a signal strength index below 7 or significant motion artifacts were excluded from further analysis.

### OCTA image processing

**Fig 1:**
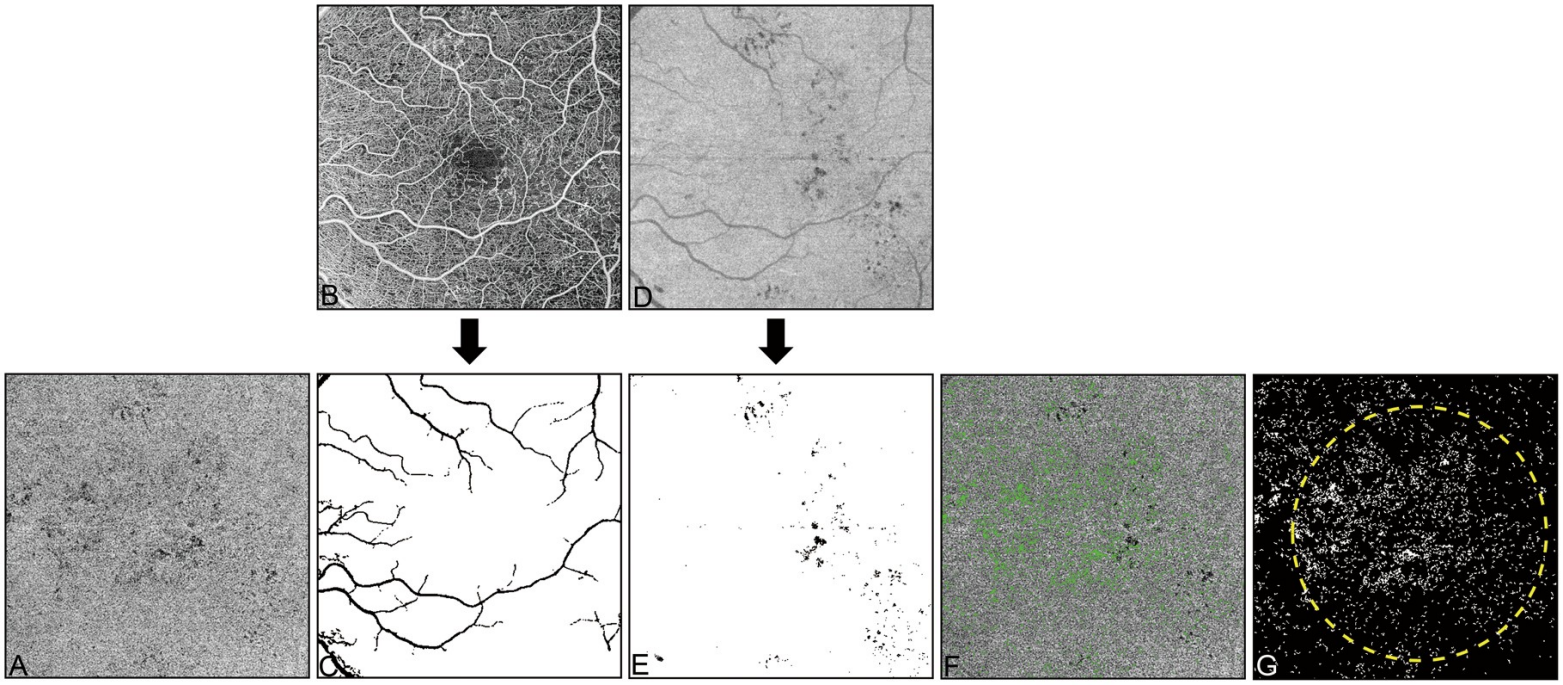
Example of image processing for the quantification of choriocapillaris flow deficits. (**A)** The *en face* choriocapillaris blood flow image without the removal of artifacts. (**B**) The *en face* retinal blood flow image. (**C**) The large retinal vessel map generated from image B. **(D**) The *en face* choriocapillaris structural image. (E) The shadowing artifacts on choriocapillaris image with black pixels corresponding to artifacts caused by retinal lesions. (**F**) The choriocapillaris flow deficits (green color) overlaid onto the *en face* choriocapillaris blood flow image (gray) after subtraction of artifacts. (**G**) The choriocapillaris flow deficit binary map for quantification. All images are from the left eye of a 45-year-old diabetic patient with proliferative diabetic retinopathy. All images are 6×6-mm fields.

Retinal and CC slabs were segmented using a validated semi-automated segmentation method [17]. The CC slab was defined as a 15-μm-thick slab starting from the outer boundary of the Bruch’s membrane (BM). A maximum projection was applied to the structural slab and a sum projection was applied to the flow slab to generate the *en face* CC images (Fig 1A). The *en face* CC structural image was provided to compensate for the shadowing artifacts caused by uneven illumination due to the heterogeneous optical property of the retinal pigment epithelium/Bruch’s membrane (RPE/BM) complex [8]. The regions on CC flow images (i.e. dark appearance) were excluded if the CC structural signal strength in that region was below 2 times the noise background floor (Fig 1D and 1E). The projection artifacts were removed by using the retinal vessel map (Fig 1B) generated from the flow signals between the inner limiting membrane and the outer plexiform layer. In addition, the regions that coincide with the positions of large retinal vessels (Fig 1C) in the CC flow image were also excluded. The resulting *en face* CC blood flow image was then used for final CC FD quantification. CC FDs were calculated using our previously published global thresholding approach [8], followed by removal of the FDs with a diameter smaller than 24 μm which represents speckle noises or physiologic FDs [11]. The percentage of flow deficits (FD%) was calculated by the ratio between the total area of FDs and the total area of the 5-mm-diameter circular region excluding regions with artifacts. The mean CC FD size was calculated as the total area of FDs divided by the number of FDs. All measurements were conducted in a 5-mm-diameter circle centered on the fovea (Fig 1). All images were analyzed using a custom MATLAB program (R2017b; MathWorks, Inc., Natick, MA, USA).

### Statistical analysis

Statistical analysis was performed using the commercial statistical software (IBM SPSS Statistics version 25.0; IBM Corporation, Armonk, New York, USA). Variable normality was inspected using histograms and the Shapiro-Wilk test. The Mann-Whitney *U* test was used to compare the CC FD% and the mean CC FD size between the diabetics and controls. The duration of DM and the glycated hemoglobin (HbA1c) level between the two DR groups were also compared using the Mann-Whitney *U* test. Analysis of variance with post hoc Bonferroni correction (parametric) or Kruskal-Wallis test with Bonferroni correction for pairwise comparison (nonparametric) was performed to compare age, intraocular pressure, blood pressure, CC FD%, and mean CC FD size between eyes with different stages of DR and controls. A *P* value less than 0.05 was considered statistically significant.

## Results

### Demographics and clinical characteristics

Forty-five eyes from 45 diabetic subjects with DR and 27 eyes from 27 age-matched control subjects were included in this study. DR was classified as nonproliferative DR (NPDR) in 25 eyes (55.6%), and proliferative DR (PDR) in 20 eyes (44.4%). The NPDR group included 11 eyes with mild NPDR, 6 with moderate NPDR, and 8 with severe NPDR. Baseline demographic and clinical characteristics are summarized in Table 1. The mean duration of DM was 19.6±8.3 years in the NPDR group and 22.1±13.5 years in the PDR group. There was no significant difference in the duration of DM between the two DR groups (*P* = 0.391). The mean HbA1c level was only marginally significantly different between the NPDR group and the PDR group (P = 0.049). There was no significant difference in age or intraocular pressure across all groups (*P* = 0.725 and *P* = 0.877, respectively). Systolic blood pressure and diastolic blood pressure were similar between groups (*P* = 0.998 and *P* = 0.311, respectively).

**Table 1:** Demographic and clinical characteristics of the study population.

Clinical characteristics	Control	NPDR	PDR
No. of subjects	27	25	20
Age (y)	56.4±13.3	54.4±16.4	53.5±8.8
Diabetes duration (y)	NA	19.6±8.3	22.1±13.5
HbA1c level (%)	NA	8.5± 2.0	7.4±1.2
SBP (mm Hg)	125.0±4.7	126.6±15.1	125.5±17.5
DBP (mm Hg)	74.3±3.2	75.1±11.0	75.8±10.9
BCVA (logMAR)	0.03±0.04	0.07±0.13	0.18±0.20
IOP (mm Hg)	15.4±2.4	15.8±2.5	15.7±3.0
No. of eyes that received anti-VEGF injections	0	2	10
No. of eyes that received pan-retinal photocoagulation	0	0	9

All values are shown as no. or mean±standard deviation.

NPDR, nonproliferative diabetic retinopathy; PDR, proliferative diabetic retinopathy; NA, not applicable; HbA1c, glycated hemoglobin; SBP, systolic blood pressure; DBP, diastolic blood pressure; BCVA, best-corrected visual acuity; LogMAR, logarithm of the minimum angle of resolution; IOP, intraocular pressure; anti-VEGF, anti-vascular endothelial growth factor.

### Quantitative analysis of the choriocapillaris

**Fig 2:**
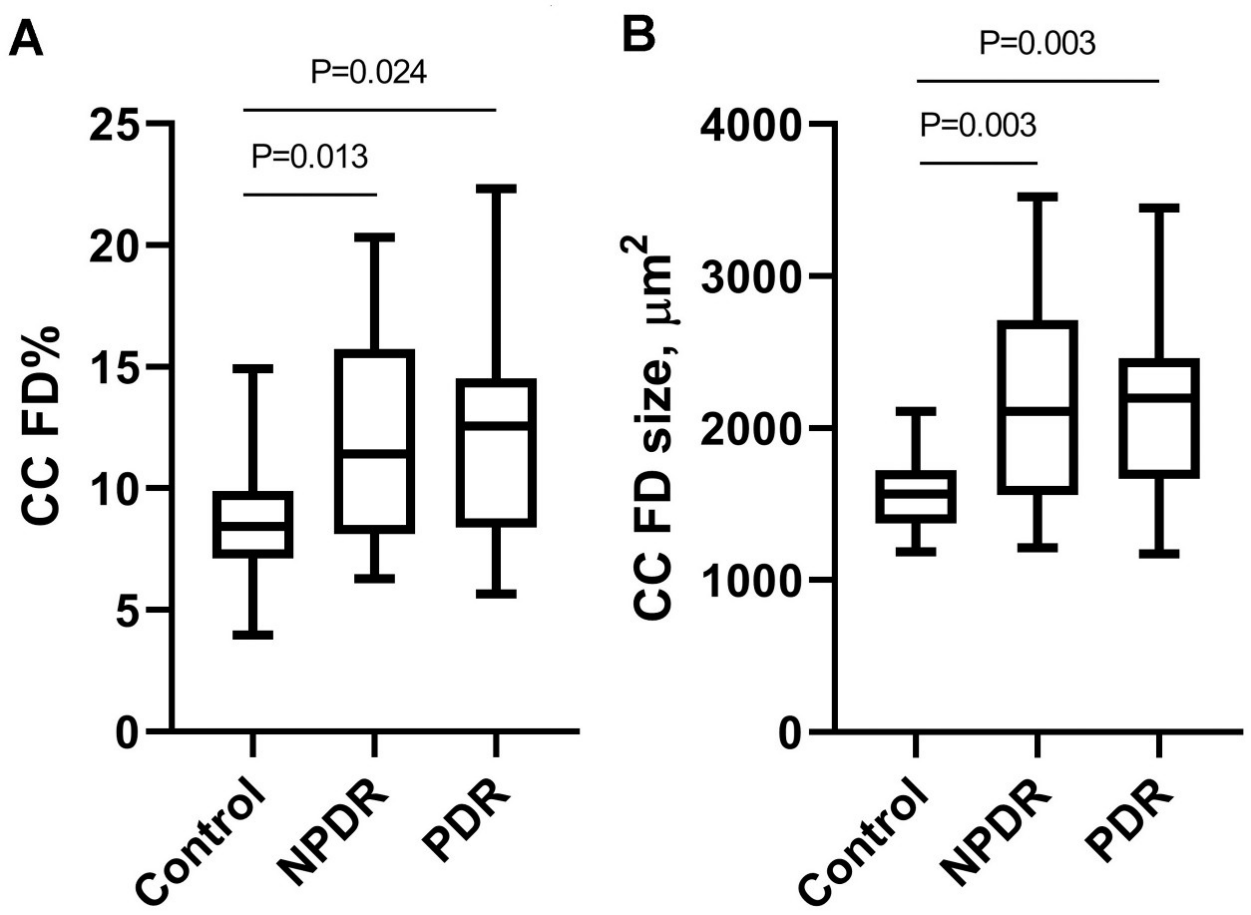
Comparisons of CC FD% (left) and mean CC FD size (right) between study groups. Kruskal-Wallis test with Bonferroni correction was performed in pairwise comparisons of the FD% and the mean FD size, respectively. Brackets indicate statistically significant differences between corresponding study groups. CC, choriocapillaris; FD, flow deficit; FD%, percentage of CC FDs; NPDR, nonproliferative diabetic retinopathy; PDR, proliferative diabetic retinopathy.

**Fig 3:**
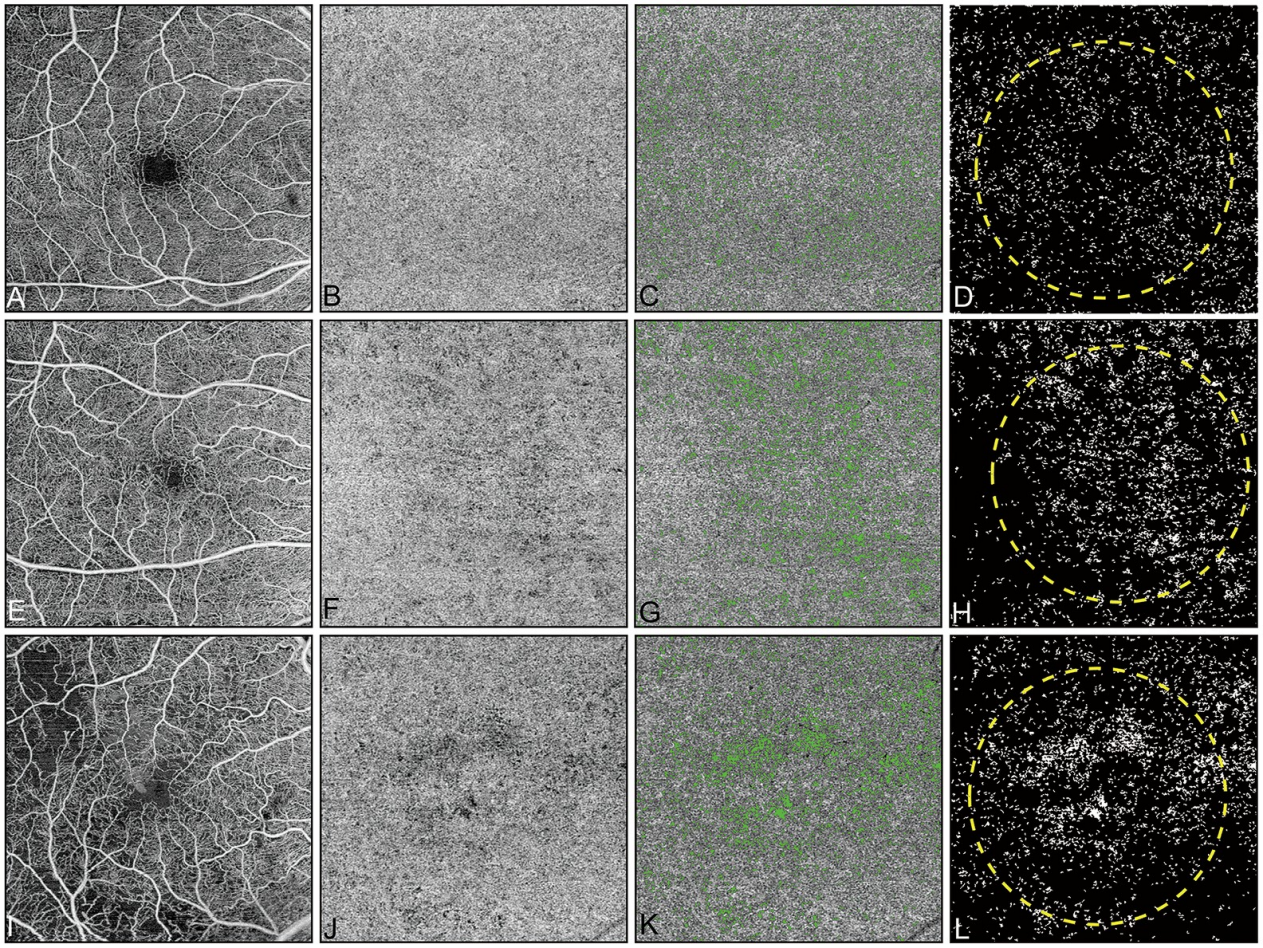
Representative illustration of the SS-OCTA images from a control eye and eyes with different stages of diabetic retinopathy. First column (A, E, and I): The *en face* retinal blood flow images. Second column (B, F, and J): The *en face* choriocapillaris blood flow images without removal of the artifacts. Third column (C, G, and K): The corresponding choriocapillaris flow deficits (green color) overlaid onto the *en face* choriocapillaris blood flow images (gray) after removal of the artifacts. Fourth column (D, H, and L): The choriocapillaris flow deficit binary maps for quantification. (A–D) Images are from the right eye of a 62-year-old healthy subject. (E–H) Images are from the right eye of a 62-year-old patient with nonproliferative diabetic retinopathy. (I–L) Images are from the right eye of a 60-year-old patient with proliferative diabetic retinopathy. All images are 6×6-mm fields).

The CC FD% in diabetic eyes was on average 1.4-fold greater than in control eyes (12.17±4.30% vs 8.82±2.61%, *P* = 0.001). The mean CC FD size in diabetic eyes was on average 1.4-fold larger than in control eyes (2151.3±650.8 μm^2^ vs 1574.4±255.0 μm^2^, *P* < 0.001). Subgroup analysis further showed that both the CC FD% (12.23±4.29% vs 8.82±2.61%, *P* = 0.013) and the mean CC FD size (2159.8±706.0 μm^2^ vs 1574.4±255.0 μm^2^, *P* = 0.003) were significantly increased in the NPDR group compared with the control group (Fig 2). Similarly, both the CC FD% (12.09±4.41% vs 8.82±2.61%, *P* = 0.024) and the mean CC FD size (2140.8±592.6 μm^2^ vs 1574.4±255.0 μm^2^, *P* = 0.003) in the PDR group were significantly increased compared with the control group (Fig 2). No significant difference in CC FD% (12.23±4.29% vs 12.09±4.41%, *P* = 1.000) or mean CC FD size (2159.8±706.0 μm^2^ vs 2140.8±592.6 μm^2^, *P* = 1.000) was observed between two DR groups. Representative *en face* images of the retinal blood flow, CC blood flow, CC flow overlaid with FDs, and binarized CC FDs with overlaid grid from a control eye and eyes with different stages of DR are shown in Fig 3.

## Discussion

The current study used a commercially available SS-OCTA system and validated algorithms to quantitatively assess the CC perfusion status in subjects with different stages of DR. Both the CC FD% and the mean CC FD size in the macular region were increased in eyes with DR.

Historically, investigations of CC alteration in diabetic eyes were limited to post-mortem histopathologic studies since the fine, fenestrated CC vasculature cannot be easily visualized by imaging modalities such as ICGA or FA [18–23]. The earliest description of CC abnormalities in diabetic eyes was from the observation of eight post-mortem or enucleated eyes with advanced PDR [16]. In these eyes, significantly narrowed CC lumina, widened inter-capillary spaces, and extensive CC dropouts were observed throughout the choroid [18]. Subsequent histopathologic studies further confirmed these angiopathic changes in CC in post-mortem eyes with NPDR [19]. The widened inter-capillary spaces and CC dropouts observed in histopathologic studies will manifest as CC FDs when imaged with OCTA. The current study corroborated the histopathologic findings, demonstrating increased CC FDs in diabetic eyes with non-invasive SS-OCTA. Moreover, CC FDs were quantitatively assessed in vivo, showing that the CC FD% was on average 1.4-fold greater, and the mean CC FD size was on average 1.4-fold larger in eyes with DR than in control eyes.

Previously, CC blood flow in diabetic eyes has been explored with spectral-domain OCTA (SD-OCTA) imaging [24, 25]. Using a commercial available SD-OCTA instrument, Conti et al. reported decreased macular CC density in eyes with DR [24]. In their study, CC density was obtained from the manufacturer’s built-in software, which is designed for the quantification of retinal vasculature but not CC vasculature. As we know, the capillary feature and distribution in retinal vasculature are significantly different from those in CC [3, 26, 27]. Compared with an averaged intercapillary distance (ICD) of 70 μm in the retinal vasculature [26], the ICDs in macular CC are only 5–20 μm [27], which is even below the lateral resolution limit (15–20 μm) of the current commercial OCTA instruments. Therefore, individual CC vessels under the macula can not be resolved by current commercial SD-OCTA. Instead of quantifying CC density directly, many researchers have opted for CC FD (or flow void) to assess CC perfusion since they are resolvable and measurable with current OCTA [6–8, 10].

Using SD-OCTA imaging, Nesper et al. also calculated the percent area of CC nonperfusion, which is similar to the CC FD% in the current study [25]. The authors reported an increased percent area of CC nonperfusion in diabetic eyes compared with control eyes. In their study, however, the authors used the pixel value from the retinal capillary layer to segment the nonperfused areas in the CC layer. Since the CC layer has a higher noise floor than the retinal capillary layer due to the scattering effect from the RPE/BM complex, applying the pixel value from the retinal capillary layer limits the interpretation of the CC data. Moreover, all the data were acquired with a SD-OCTA instrument, which has limited capability for CC visualization due to its relatively short wavelength (central wavelength at approximately 840 nm).

The SS-OCTA system utilizes a longer wavelength (central wavelength at approximately 1060 nm), which reduces scattering from the RPE/BM complex, allowing for deeper light penetration into the CC [28]. Using an investigational SS-OCTA system, Choi et al. observed the macular CC perfusion in normal eyes and diabetic eyes without quantitative analysis [29]. Compared with a homogeneous pattern in normal CC, focal or diffuse CC flow impairment was observed in 15 of the 29 eyes with NPDR and 7 of the 9 eyes with PDR [29]. One limitation of their study was that the subjects with DR were on average more than ten years older than the normal subjects, which may have introduced bias since CC flow impairment increases with age [6, 7]. Using SS-OCTA, Gendelman et al. found that CC FD% in DM was positively associated with age and DR severity [30]. In their study, however, shadowing artifacts caused by retinal lesions were not excluded before CC quantification, which could potentially confound CC FD measurements since retinal lesions in eyes with DR may increase with DR severity [14]. In the current study, we quantitatively analyzed the macular CC perfusion with a commercial available SS-OCTA system in diabetic eyes as well as healthy eyes, while controlling for variables such as age and extremes in axial length. Moreover, uncompensated shadowing artifacts from retinal lesions were also excluded before CC quantification to ensure accurate measurement of the true FDs in eyes with DR. We believe the current study provides stronger evidence for the CC perfusion status in DR than the previous studies.

No significant difference in the CC FD% or the mean CC FD size was observed in the macular region between eyes with NPDR and eyes with PDR. This may be related to the complicated microangiopathic alterations in CC as DM progresses. Using scanning electron microscopy, Fryczkowski et al. observed the simultaneous appearance of CC luminal narrowing in one capillary and dilation in another in post-mortem eyes from subjects with advanced DM [21, 23]. Frequent formations of microaneurysms and vascular loops in CC were also observed in these eyes [21, 23]. Therefore, on the one hand, narrowed CC lumina and CC dropouts increased in DR, which resulted in enlarged CC FD size and increased CC FD% on OCTA images. On the other hand, capillary dilations, microaneurysms, and CC vascular loops also occurred with DR progression, which might result in reduced CC FD size and decreased CC FD%. Due to the limited resolution of current commercial OCTA instruments, these subtle morphological alterations in individual CC can not be differentiated from the densely packed CC vasculature under the macula. However, from analyses for the CC FDs, the macular CC perfusion seems to keep relatively constant despite the progression of DR. The retinal vasculature and choroidal vasculature are two separate vascular systems with different hierarchies, hemodynamic properties, and regulatory mechanisms [3, 31, 32]. Our results suggest that the CC involvement in eyes with DR may have unique characteristics that are less correlated with clinical DR severity, at least in the macular region.

We acknowledge several limitations to this study. First, subtle morphological alterations in individual CC under the macula can not be resolved by current OCTA systems, which limits further understanding of the microangiopathic alterations in diabetic choroids. With the evolution of OCTA technology, clearer visualization of the CC vasculature will help us understand this important vascular layer better. Second, our study included a relatively small number of subjects. Excluding subjects with other systemic and ocular diseases that may affect ocular circulation greatly limited the number of subjects we were able to enroll in this study, but the strict exclusion criteria also reduced the number of confounding variables. Future studies with larger sample sizes are needed to confirm these findings. Third, although we have excluded patients with recent therapeutic interventions, prior therapeutic interventions may have affected the study outcomes in some previously treated patients. Further longitudinal studies with larger sample sizes are needed to investigate the effects of therapeutic interventions on CC blood flow in diabetic eyes. Finally, we only analyzed CC perfusion in the central 5-mm-diameter circular region. Although CC vasculature loss in diabetic eyes has been demonstrated to be more prominent in the posterior pole than in the periphery [20], CC perfusion in the peripheral choroid should also be investigated in future studies.

In summary, the current study demonstrates the ability of non-invasive SS-OCTA to capture and quantitate CC blood flow in diabetic eyes. Both CC FD% and CC FD size in the macular region are increased in eyes with DR. SS-OCTA provides new insights into the in vivo investigation of the CC perfusion status in diabetic eye disease as well as other ocular vascular diseases.

## Supporting information

S1 Table (XLSX)
